# Development of a Novel Ultrasound-guided Peritonsillar Abscess Model for Simulation Training

**DOI:** 10.5811/westjem.2017.11.36427

**Published:** 2017-12-14

**Authors:** Vivienne Ng, Jennifer Plitt, David Biffar

**Affiliations:** * University of Arizona, Department of Emergency Medicine, Tucson, Arizona; † University of Arizona, Arizona Simulation Technology & Education Center, Tucson, Arizona

## Abstract

**Introduction:**

Peritonsillar abscess (PTA) is the most common deep space infection of the head and neck presenting to emergency departments.^1^ No commercial PTA task trainer exists for simulation training. Thus, resident physicians often perform their first PTA needle aspiration in the clinical setting, knowing that carotid artery puncture and hemorrhage are serious and devastating complications. While several low-fidelity PTA task trainers have been previously described, none allow for ultrasound image acquisition.^6^–^9^ We sought to create a cost-effective and realistic task trainer that allows trainees to acquire both diagnostic ultrasound and needle aspiration skills while draining a peritonsillar abscess.

**Methods:**

We built the task trainer with low-cost, replaceable, and easily cleanable materials. A damaged airway headskin was repurposed to build the model. A mesh wire cylinder attached to a wooden base was fashioned to provide infrastructure. PTAs were simulated with a water and lotion solution inside a water balloon that was glued to the bottom of a paper cup. The balloon was fully submerged with ordnance gelatin to facilitate ultrasound image acquisition, and an asymmetric soft palate and deviated uvula were painted on top after setting. PTA cups were replaced after use. We spent eight hours constructing three task trainers and used 50 PTA cups for a total cost <$110.

**Results:**

Forty-six emergency medicine (EM) residents performed PTA needle aspirations using the task trainers and were asked to rate ultrasound image realism, task trainer realism, and trainer ease of use on a five-point visual analog scale, with five being very realistic and easy. Sixteen of 46 (35%) residents completed the survey and reported that ultrasound images were representative of real PTAs (mean 3.41). They found the model realistic (mean 3.73) and easy to use (mean 4.08). Residents rated their comfort with the drainage procedure as 2.07 before and 3.64 after practicing on the trainer.

**Conclusion:**

This low-cost, easy-to-construct simulator allows for ultrasound image acquisition while performing PTA needle aspirations and is the first reported of its kind. Educators from EM and otolaryngology can use this model to educate inexperienced trainees, thus ultimately improving patient safety in the clinical setting.

## BACKGROUND

Peritonsillar abscess (PTA) is the most common deep space infection of the head and neck presenting to emergency departments (ED).^1^ Draining a PTA is straightforward and can be accomplished with needle aspiration, incision and drainage, or tonsillectomy. With a cure rate of 93–95%,^1^ needle aspiration is the most common approach, does not require special equipment, and is relatively simple and inexpensive. However, physical exam alone has not been shown to reliably differentiate between PTA and cellulitis, and blind needle aspiration has a reported false negative rate of 10–24%.^2^–^3^ The addition of intraoral ultrasound can improve diagnosis (sensitivity 89–95%; specificity 79–100%)^4^ and aid in the safe performance of needle aspiration. In a prospective, randomized, controlled trial comparing the diagnostic accuracy of emergency providers for detecting PTA or cellulitis using intraoral ultrasound or landmark technique, ultrasound established the correct diagnosis more often and led to more successful aspiration of purulent material than landmark technique. Additionally, the average number of needle punctures was lower in the ultrasound than landmark group.^5^

Currently, no commercial PTA task trainer exists for simulation training, and thus resident physicians often perform their first PTA needle aspiration in the clinical setting, with the knowledge that carotid artery puncture and hemorrhage are serious and devastating complications. Thus, simulating PTA needle aspirations with a realistic model that allows for ultrasound image acquisition and procedural competence can build confidence and proficiency prior to performing this procedure in patient care. While several low-fidelity PTA task trainers have been previously described,^6^–^9^ none allow for ultrasound-guided diagnosis and management, which is the preferred strategy in the ED setting for patient safety and comfort, and all have limitations ranging from ease and stability of construction to anatomic fidelity.

## OBJECTIVES

We sought to create a cost-effective and realistic task trainer that allows trainees to acquire both diagnostic ultrasound and needle aspiration skills while draining a PTA.

## CURRICULUAR DESIGN

We built the task trainer with low-cost, replaceable, and easily cleanable materials. A damaged Laerdal^©^ Adult Airway Management Trainer headskin with airways, teeth, and naturally occurring trismus was repurposed to build the PTA model. We fashioned a mesh wire cylinder attached to a wooden base to provide internal structure and access to the posterior oropharynx. PTAs were simulated with a water and dimethicone barrier lotion solution inside a thin latex water balloon that was glued to the bottom of a paper cup, as described by Bunting et al.^3^ The balloon was fully submerged with Vyse Ordnance Gelatin (pork gelatin/hydrolysate) to facilitate ultrasound image acquisition. An asymmetric soft palate and deviated uvula were painted on top of the gelatin mold after it was allowed to set in a refrigerator overnight. PTA cups were replaced after each successful needle aspiration, as water balloons only tolerate one needle puncture each.

Materials required can be purchased from a hardware store or Amazon (Table). Detailed construction instructions follow. A complete pictorial guide can be found at http://escholarship.org/uc/uciem_westjem (See Supplement).

### Construction

**Figure 1:**
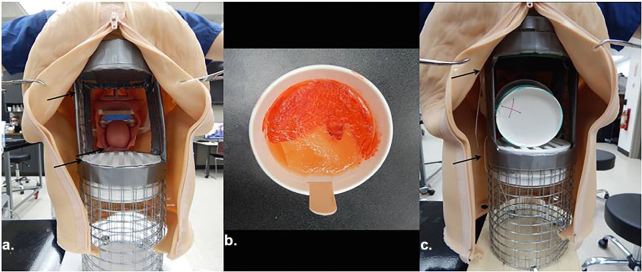
(a) Supportive mesh cylinder from behind; (b) Finished peritonsillar abscess cup; (c) Peritonsillar abscess cup inserted into task trainer.

1. Prepare the headskin. Headskins with teeth and a tongue that are ready to be discarded from any airway task trainer or mannequin can be used.
  - From the inside of the headskin, remove the trachea, bronchi, and any other anatomic parts until only the tongue and internal frame remain. Cut a slit at the base of the tongue to allow a craft stick to be slotted in and for zip-tie attachment to internal frame.
2. Prepare the supportive stand (Figure 1a). The purpose is to provide structure to the floppy headskin while also allowing easy access to the posterior oropharynx through the mouth for the trainee and from the back for the facilitator.
  - Cut hardware cloth to the height needed* to support the head in an upright position. Wrap hardware cloth around one 4” PVC sewer and drain fitting* to ensure fit. Two layers of cloth are suggested for added stability. Secure the mesh cylinder with the surplus bailing wire packaged with the hardware cloth.
  - Using wire cutters, remove a posterior portion of the cylinder wide and high enough* for a facilitator to place and hold a PTA cup at the level of the mouth opening. Similarly, remove an anterior portion* of the cylinder to allow easy access to the PTA cup through the mouth. To prevent injury, use duct tape to cover the exposed metal edges.
  - Position the 4” PVC sewer and drain fitting at the level of the base of the tongue*, aligning with the inferior aspect of the posterior and anterior openings, thus creating a platform on which the PTA cup is placed. Zip tie the drain fitting in place to the cylinder. Cap the top of the cylinder with one NDS 4” drain grate* to provide support to the top of the head and prevent bowing. Zip tie in place.
  - Affix the cylinder to scrap plywood using utility hook hangers and screws, or similar. Secure the cylinder to the headskin’s internal frame with zip ties at multiple points, including through the slit made at the base of the tongue. This is crucial to maintaining an upright and anterior position of the PTA cup when the mouth is opened by the learner during the procedure. If desired, fill the cranial space above the cylinder with scrap foam or towels to provide structure to the head.
3. Prepare the peritonsillar abscess (Figure 1b).
  - Combine water and dimethicone barrier lotion to desired viscosity. Ensure simulated abscess material can be aspirated through an 18g spinal needle. Inject approximately 7ml of material into a small water balloon and tie closed.
  - Glue the balloon to the bottom of an 8oz paper ice cream cup with cyanoacrylate glue. Mark the location of the PTA balloon on the underside of the cup.
  - Tape half of a craft stick to the inside of the cup inferior to the abscess to ensure proper orientation in the airway head. Cover the balloon with a layer of cotton to obscure the balloon.
  - Prepare ballistic gelatin. Combine 100g of gelatin powder per 800ml of water in a glass beaker and stir. Place beaker into heated water bath of at least 75C and allow to sit for a minimum of 10 minutes while stirring occasionally. Add approximately one drop each of red and yellow food coloring per 800ml to achieve desired flesh color. Using heat protective gloves or oven mitts, pour gelatin into the cup to cover both the balloon and cotton. Refrigerate for a minimum of two hours to fully set.
  - Using red paint or moulage makeup, paint an asymmetric soft palate and deviated uvula on top of the gelatin, appropriately corresponding to the location of the balloon (Figure 1b).
4. Model completion. Insert the craft stick from the PTA cup into the slit at the base of the tongue of the airway head. This stabilizes and correctly orients the cup during needle aspiration. Additionally, the facilitator is made aware of the PTA location, as indicated by the marking previously made on the underside of the cup (Figure 1c).

Approximately three hours were invested to build three task trainers, and an additional five hours were required to make 50 PTA cups.

## IMPACT/EFFECTIVENESS

After a didactic session reviewing peritonsillar abscess presentation, treatment, and management, 46 EM residents performed PTA needle aspirations using the task trainers. Due to the airway headskin’s plastic material, naturally occurring trismus realistically simulated the difficulty in performing ultrasound-guided aspiration. Faculty instructors provided direct feedback on ultrasound technique and procedural skills.

Residents were anonymously surveyed on their comfort performing PTA needle aspiration before and after the simulation session and were asked to rate ultrasound image realism, task trainer realism, and trainer ease of use on a five-point visual analog scale (VAS), with five being very realistic and easy. Sixteen of 46 (35%) residents completed the survey. Eleven had previously drained 1–3 PTAs in clinical practice, with the remaining five having no prior experience. On the VAS, residents rated their comfort with the PTA drainage procedure as 2.07 before and 3.64 after practicing the procedure on the trainer. Residents found that ultrasound images were representative of real PTAs (mean 3.41, range 2.4–4.7). They also reported that the task trainer was realistic (mean 3.73, range 2.5–4.8) and easy to use (mean 4.08, range 1.0–5.0).

**Figure 2:**
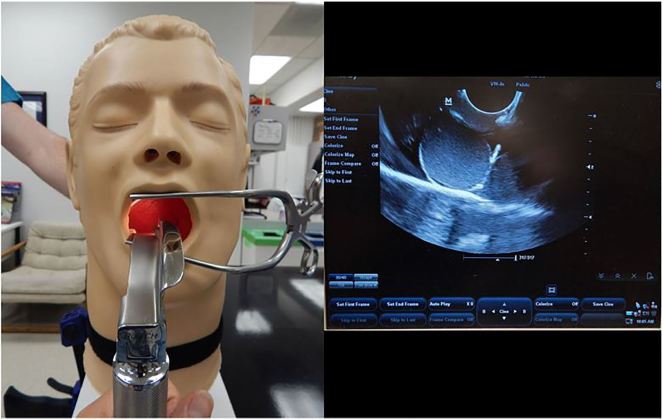
Completed ultrasound-guided peritonsillar abscess task trainer and corresponding ultrasound image.

Our model, based upon that of Bunting et al., has several advantages over previous trainers including the incorporation of pork gelatin/hydrolysate to facilitate realistic ultrasound image acquisition with a fair and differentiable interface between the abscess and oropharynx (Figure 2) and improvements to the headskin infrastructure with the addition of a sturdy mesh cylinder and plywood base, allowing it to be mounted securely to any table. The improved stability allows the model to tolerate significant manipulation during the simulated procedure, including the forces applied to overcome trismus and used with an intraoral ultrasound probe while acquiring images of the abscess. The abscess itself is also compressible, and needle visibility and echogenicity are similar to that in real practice. Despite the time required to create this infrastructure, costs remain low; and once built, the task trainer can be adapted for different otolaryngologic procedures such as epistaxis management, tongue laceration repair, and post-tonsillectomy bleeding management. PTA cups can be made on demand for any learner group size.

## LIMITATIONS

Our innovation has several limitations. The primary capital cost to construct the task trainer and abscess cups is time and experience. Physicians and non-healthcare providers built our trainers. Thus, it would be reasonable to suggest that any simulation technology specialist with at least one year of experience has the skills to build the model. If no discarded headskin is available, a new one costs upwards of $1,000. Our model used a Laerdal^©^ headskin. While any discarded headskin should be adaptable to this trainer, others were not tested. Acquired ultrasound images are rudimentary and limited in their anatomic fidelity due to lack of adjacent tonsillar tissue and carotid artery, difficulty simulating abscess heterogeneity including septations and locations, and presence of needle tracks with multiple attempts. Additionally, cup edges are hyperechoic and may detract from the fidelity of the soft-tissue image.

Given the rudimentary images, validity evidence of the trainer’s ability to teach accurate PTA diagnosis was not pursued. With regard to simulation implementation, the number of learners that can be trained and assessed is dependent on the number of simultaneously available task trainers and facilitators. Our survey response rate was low and limited by resident willingness to complete, thus prohibiting any meaningful statistical analysis. Furthermore, we surveyed novice learners on subjective constructs, a third of whom had never performed a PTA aspiration. This limits the impact and generalizability of their responses and our findings of improved procedural comfort, which is to be expected after a simulation experience. Finally, we did not pursue independent validation of the task trainer for the needle aspiration procedure, as our model was based upon that of Bunting et al, who validated their trainer with senior resident and attending otolaryngologists for both needle aspiration and incision-and-drainage techniques.^6^

## CONCLUSION

In summary, we developed a low-cost, do-it-yourself, and easy-to-construct simulator that allows for ultrasound image acquisition while performing peritonsillar abscess needle aspirations. Our trainees found the ultrasound images realistic and had increased understanding of and comfort with needle aspiration management after practicing on the model. To the best of our knowledge, we report the first ultrasound-guided peritonsillar abscess model for simulation training. Educators from EM and otolaryngology can use this model to educate inexperienced trainees, thus ultimately improving patient safety in the clinical setting. Future work on the trainer should focus on improving ultrasound image fidelity to include diagnostic characteristics felt important by the ultrasound community.

## Supplementary Information



## Figures and Tables

**Table:** Materials for peritonsillar abscess model.

3 Task trainer heads and internal support		100 Peritonsillar abscess cups (replaceable)	
	
Total cost	$46	Total cost	$59
Discarded headskin	$0 each	Dimethicone barrier lotion	$10
Hardware cloth	$16	Water balloons (500 ct)	$5
PVC sewer and drain fitting, 4″ outer diameter	$3.50 each	Craft sticks (100 ct)	$5
NDS drain grate, 4″ inner diameter	$2.80 each	8oz paper ice cream cups (100 ct)	$17
Duct tape	$3.50	Cyanoacrylate glue	$3
Zip ties	$3	600oz ballistic gelatin	$13
Utility hook hangers & screws	$4	Food coloring	$2
Scrap plywood	$0	Cotton balls/pads	$2
Scrap foam or towels	$0	Paint	$2

PVC, polyvinyl chloride; *NDS*, national drainage system.

## Footnotes

* Exact measurements and positioning are specific to the headskin used and are determined by trial and error.

## References

[1] Riviello RJ, Roberts JR, Custalow CB, Thomsen TW, Hedges JR (2014). Otolaryngologic Procedures. Roberts and Hedges’ Clinical Procedures in Emergency Medicine.

[2] Prokofieva A, Modayil V, Chiricolo G (2016). Ultrasound-guided drainage of peritonsillar abscess: shoot with your hockey-stick. Intern Emerg Med.

[3] Lyon M, Blaivas M (2005). Intraoral ultrasound in the diagnosis and treatment of suspected peritonsillar abscess in the emergency department. Acad Emerg Med.

[4] Hartman ND, Tintinalli JE, Stapczynski J, Ma O, Yealy DM, Meckler GD, Cline DM (2016). Neck and Upper Airway. Tintinalli’s Emergency Medicine: A Comprehensive Study Guide, 8e.

[5] Costantino TG, Satz WA, Dehnkamp W (2012). Randomized trial comparing intraoral ultrasound to landmark-based needle aspiration in patients with suspected peritonsillar abscess. Acad Emerg Med.

[6] Bunting H, Wilson BM, Malloy KM (2015). A novel peritonsillar abscess simulator. Simul Healthc.

[7] Taylor SR, Chang CW (2014). Novel peritonsillar abscess task simulator. Otolaryngol Head Neck Surg.

[8] Murphy J, Murphy JT, Sama A (2007). Quinsy trainer. J Laryngol Otol.

[9] Giblett N, Hari C (2016). Introducing a realistic and reusable quinsy simulator. J Laryngol Otol.

