# Supplementary material for: Development of a Novel Ultrasound-guided Peritonsillar Abscess Model for Simulation Training
Source: West J Emerg Med. 2017 Dec 14;19(1):172–6. doi: 10.5811/westjem.2017.11.36427 (PMC5785189; doi:10.5811/westjem.2017.11.36427)
Supplement: Supplementary file 1 [file wjem-19-172-s001.pdf]

Development of a Novel Ultrasound-guided Peritonsillar Abscess Model for Simulation Training

Supplement: Construction Instructions with Pictorial Guide

Materials for task trainer heads (see Table 1)	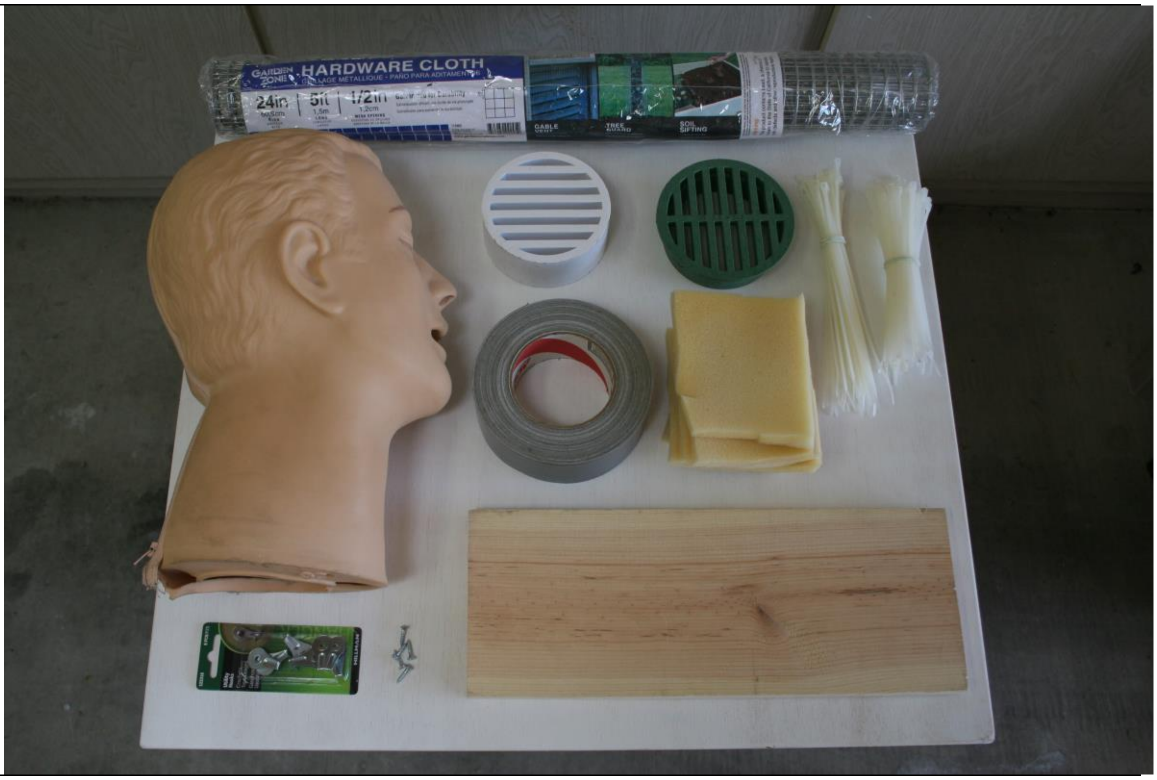

Prepare the headskin with slit at base of tongue (1a)	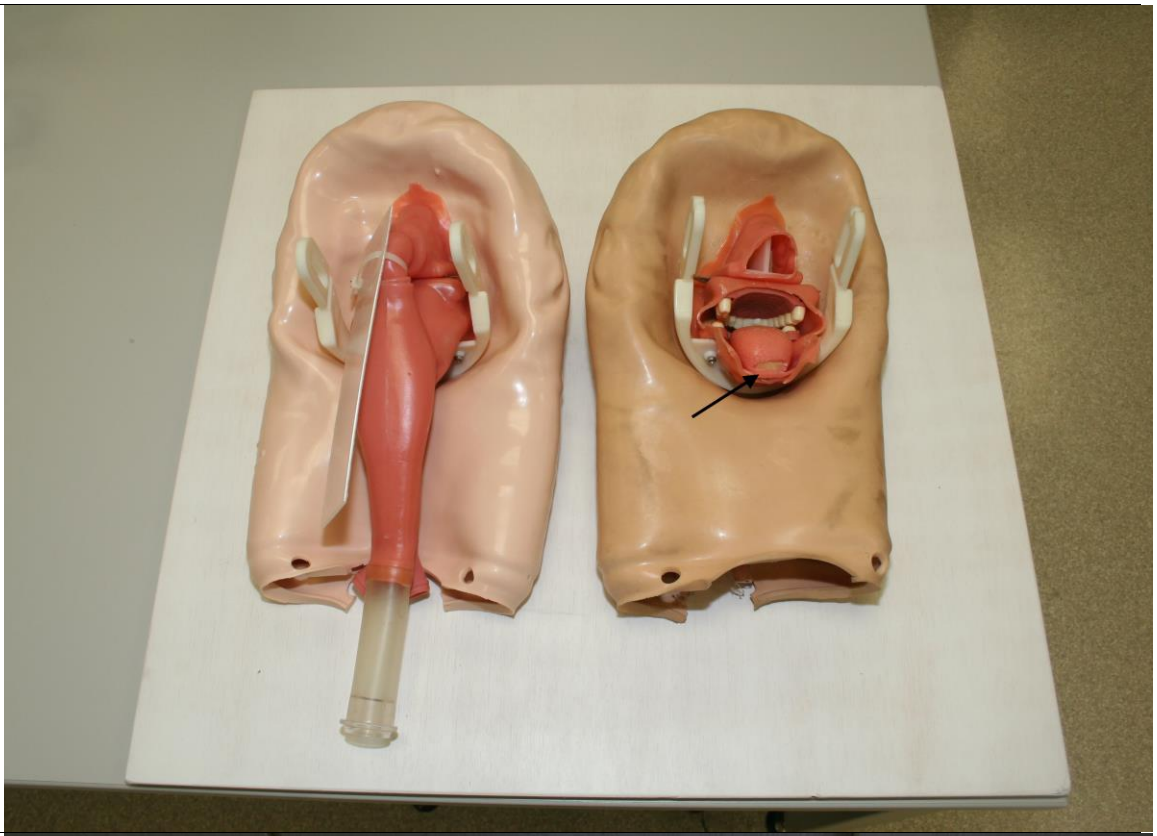
Cut hardware cloth to height needed for available headskin (2a)	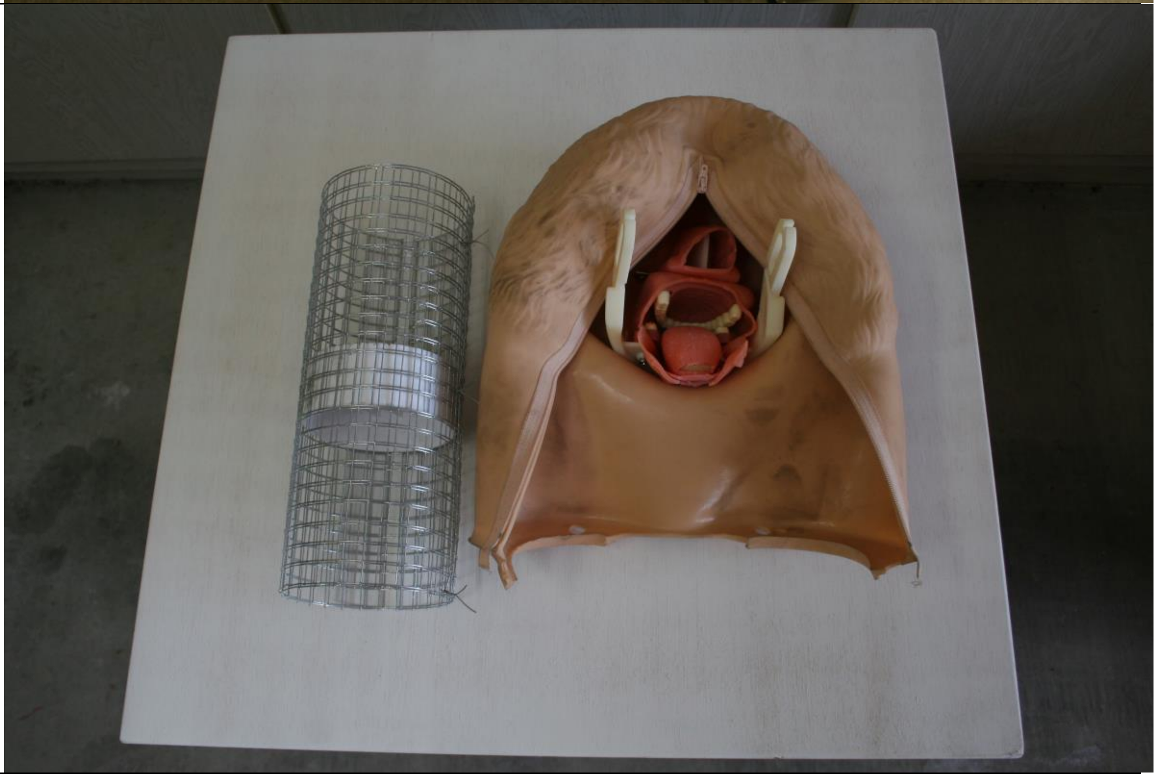

Wrap hardware cloth around PVC sewer and drain fitting. Two layers are suggested for added stability. (2a)	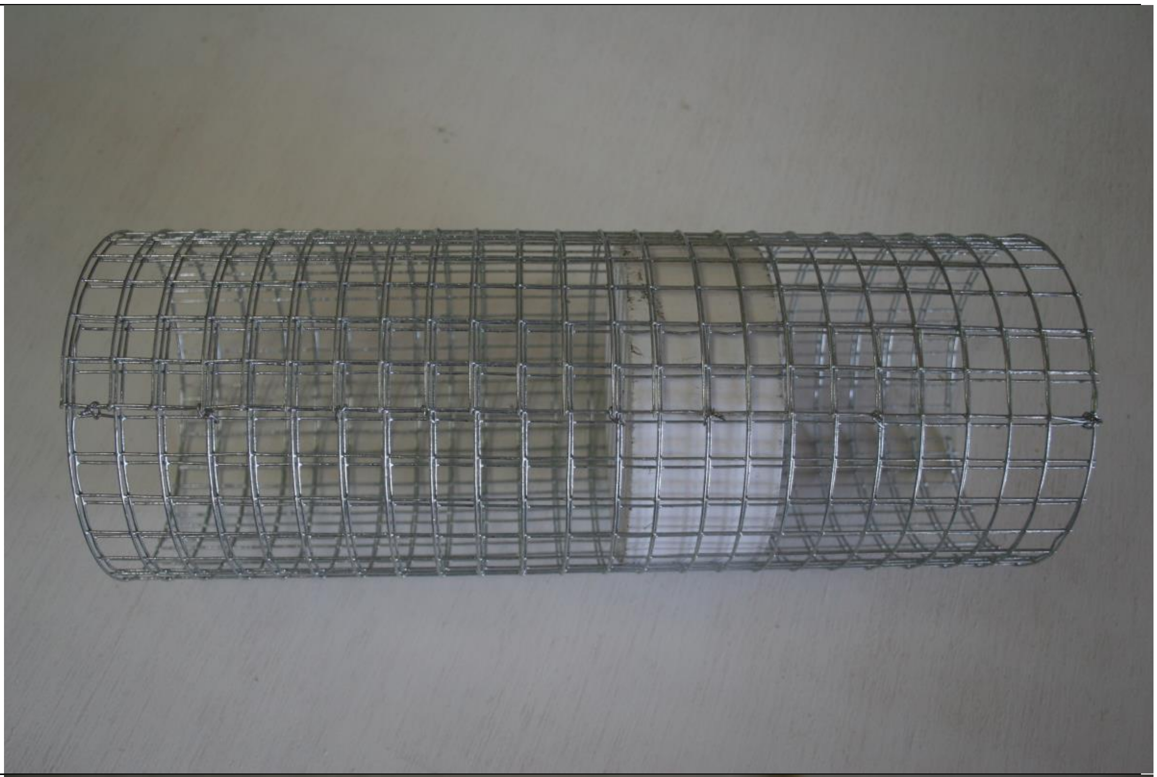
Secure cylinder with bailing wire (2a)	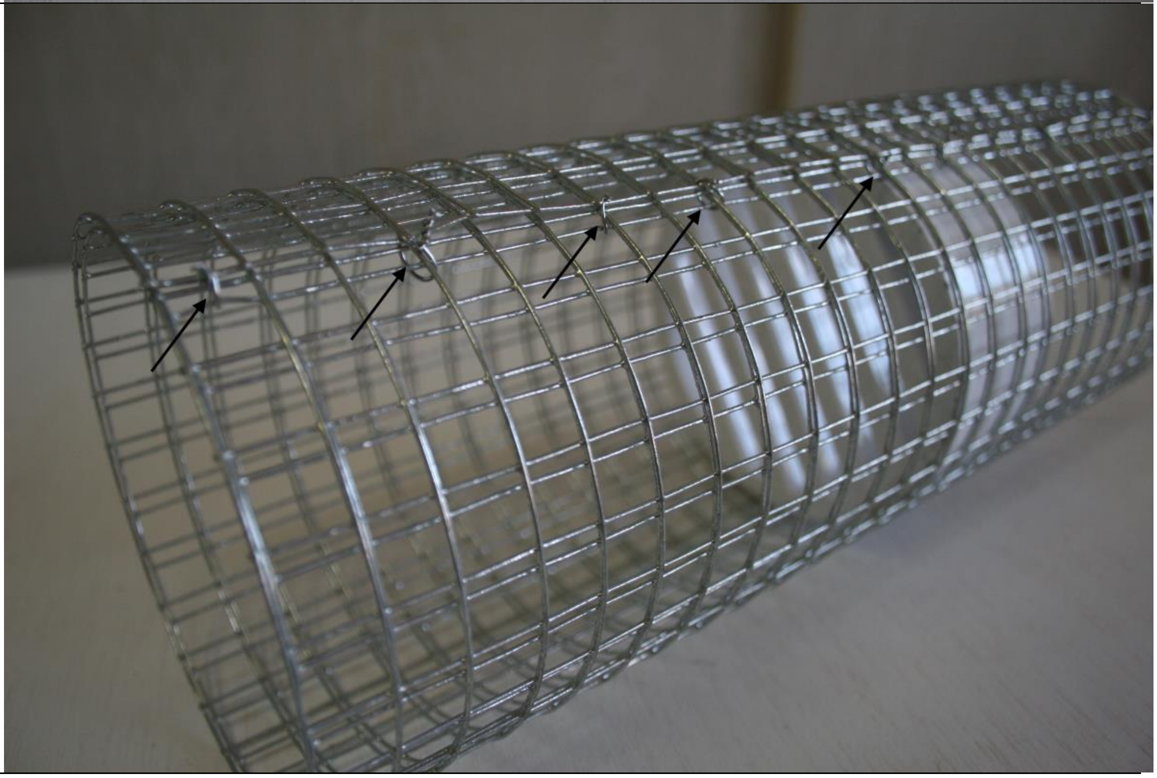

Remove posterior cylinder section. Position of opening is determined by position of mouth opening in headskin. Size of opening is determined by size of cups being used. (2b)	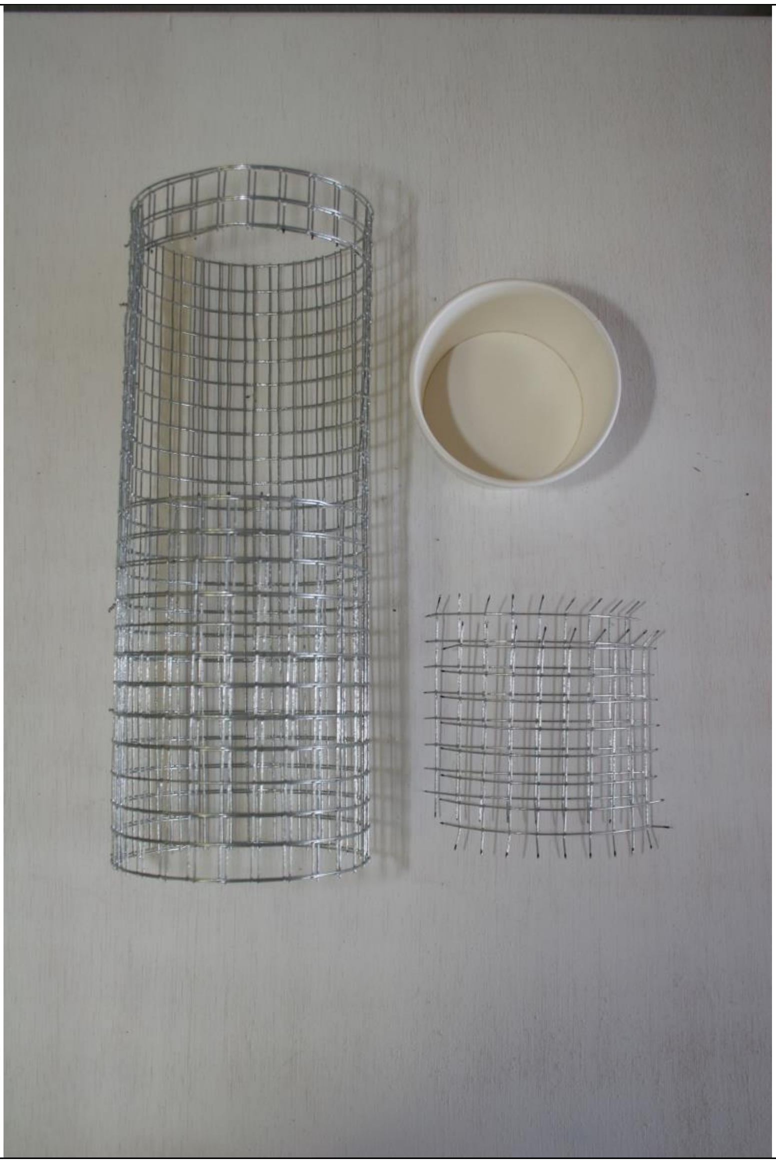

Remove anterior cylinder section (2b)		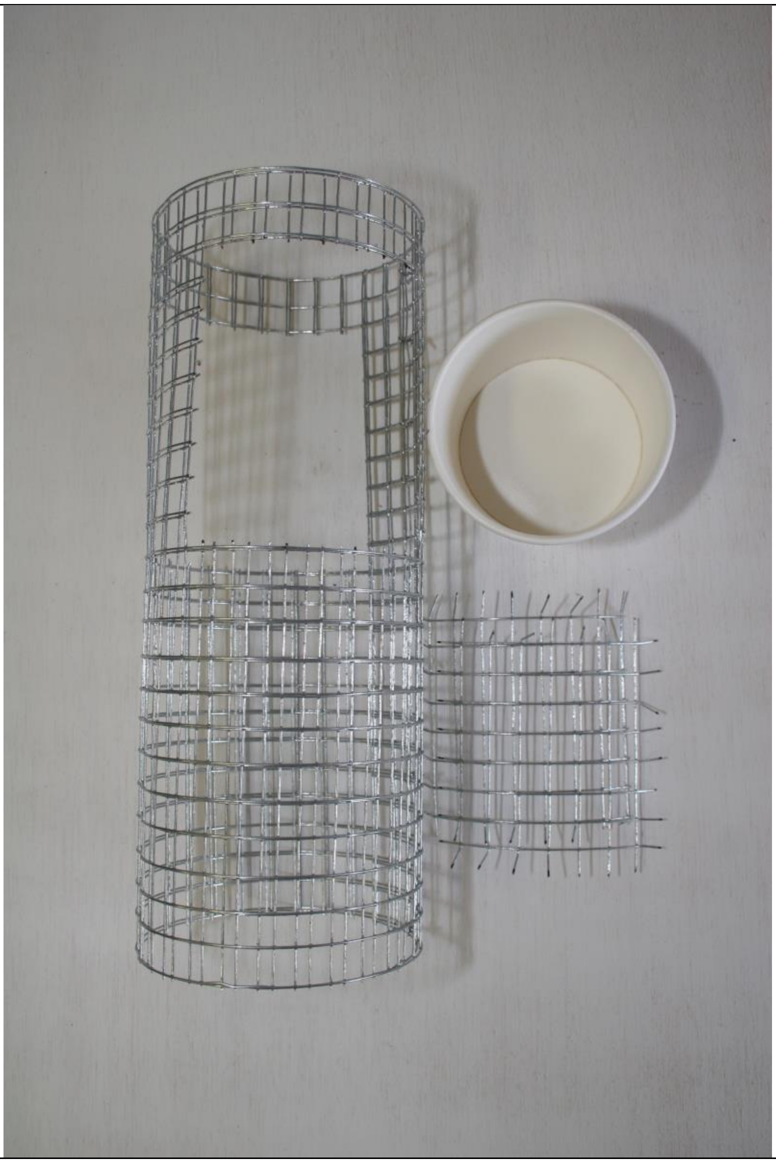

Affix duct tape to cover sharp edges (2b)	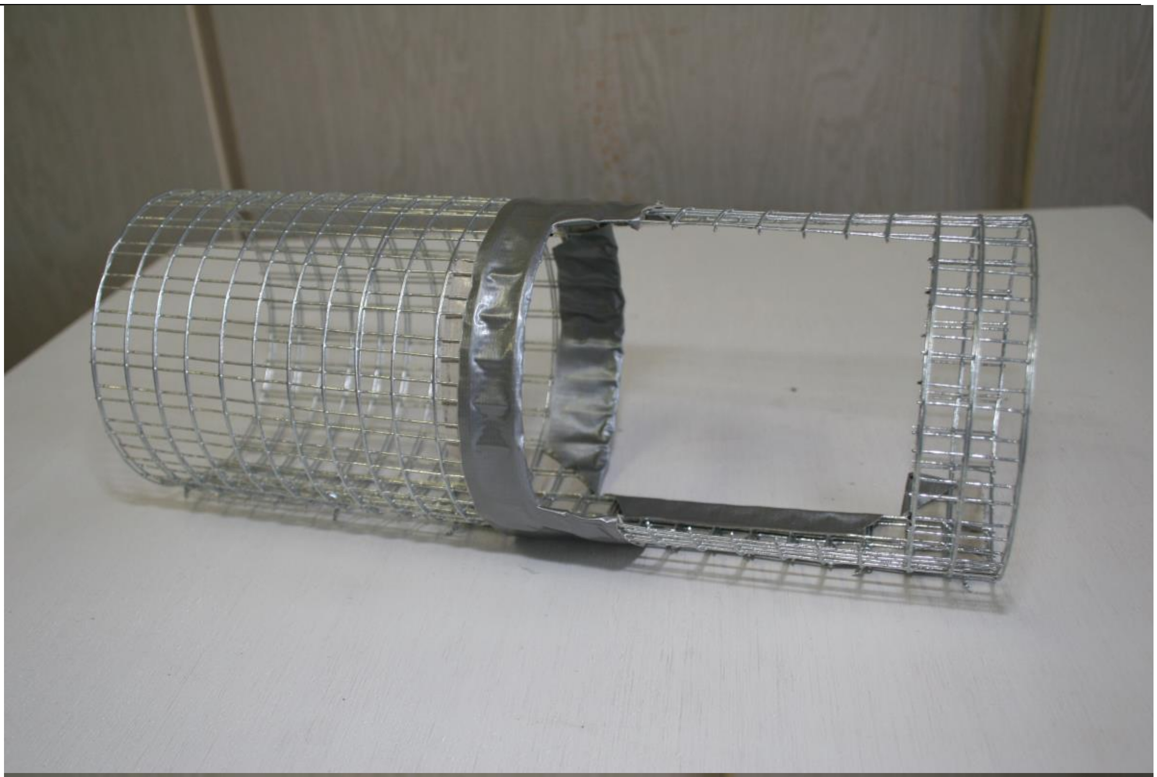 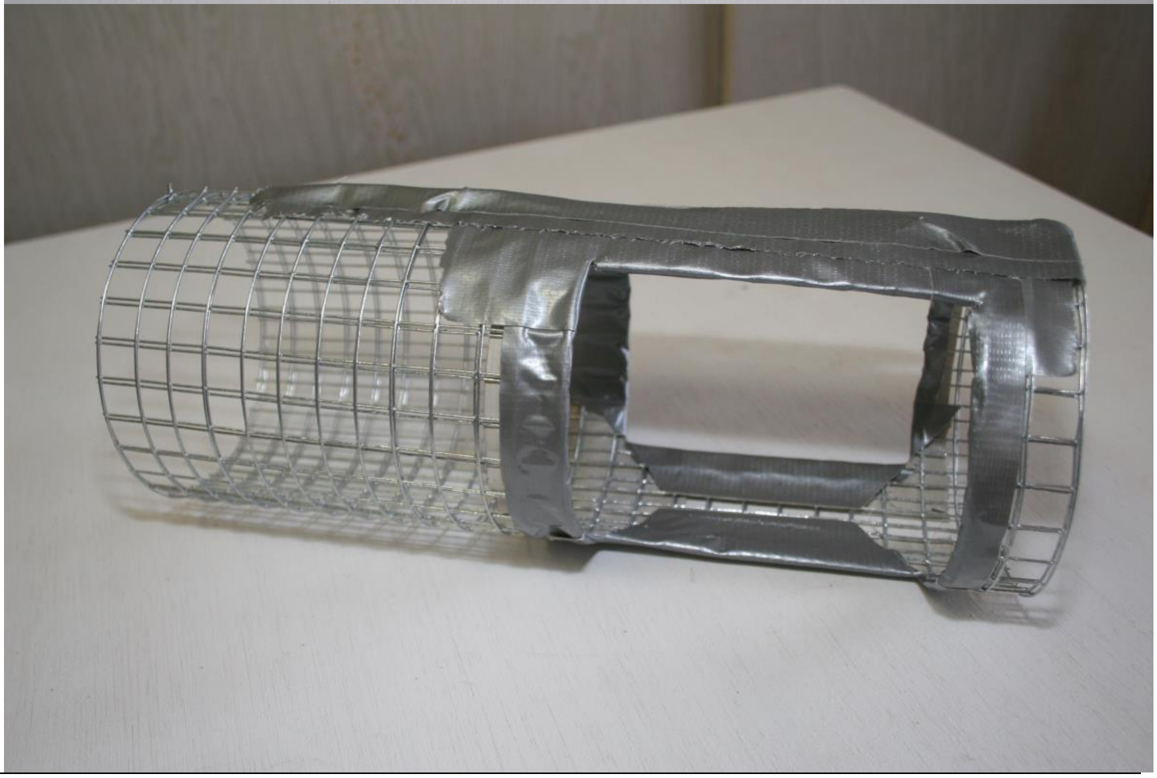

Position sewer and drain fitting, aligning with the base of the tongue, such that it can serve as a platform on which the PTA cup is placed. Secure with zip ties. (2c)	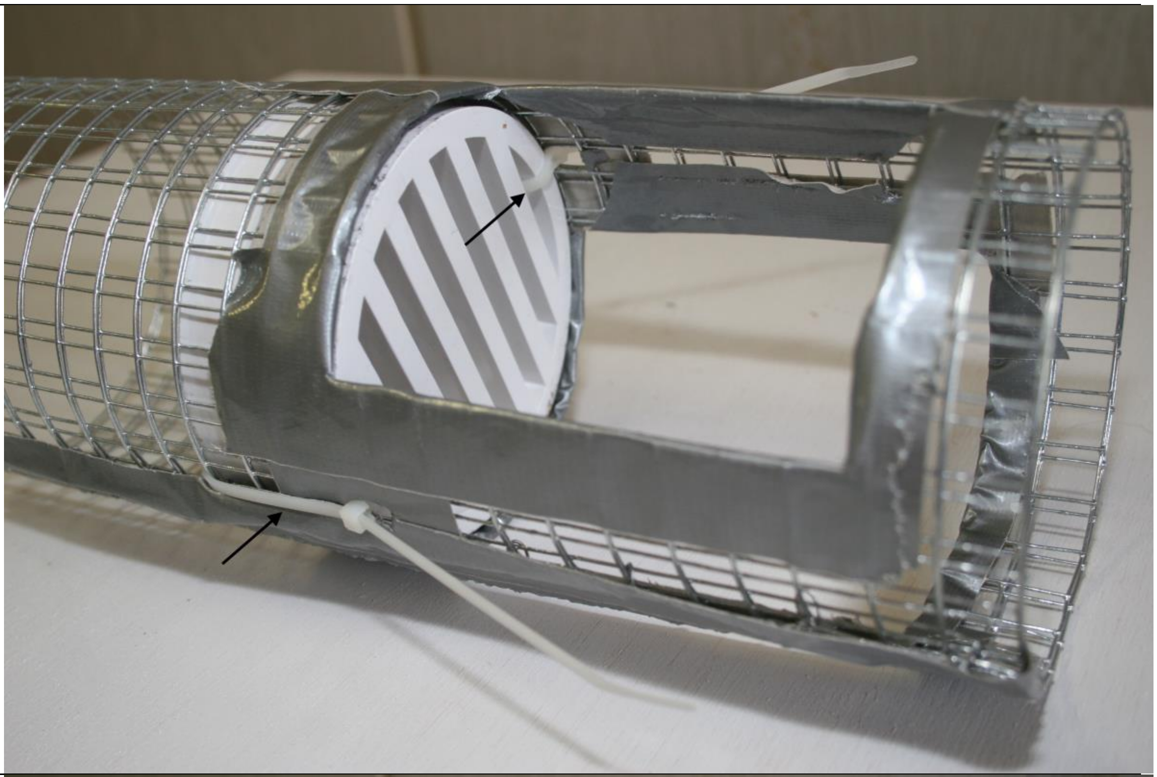
Cap with NDS drain grate and secure with zip ties (2c)	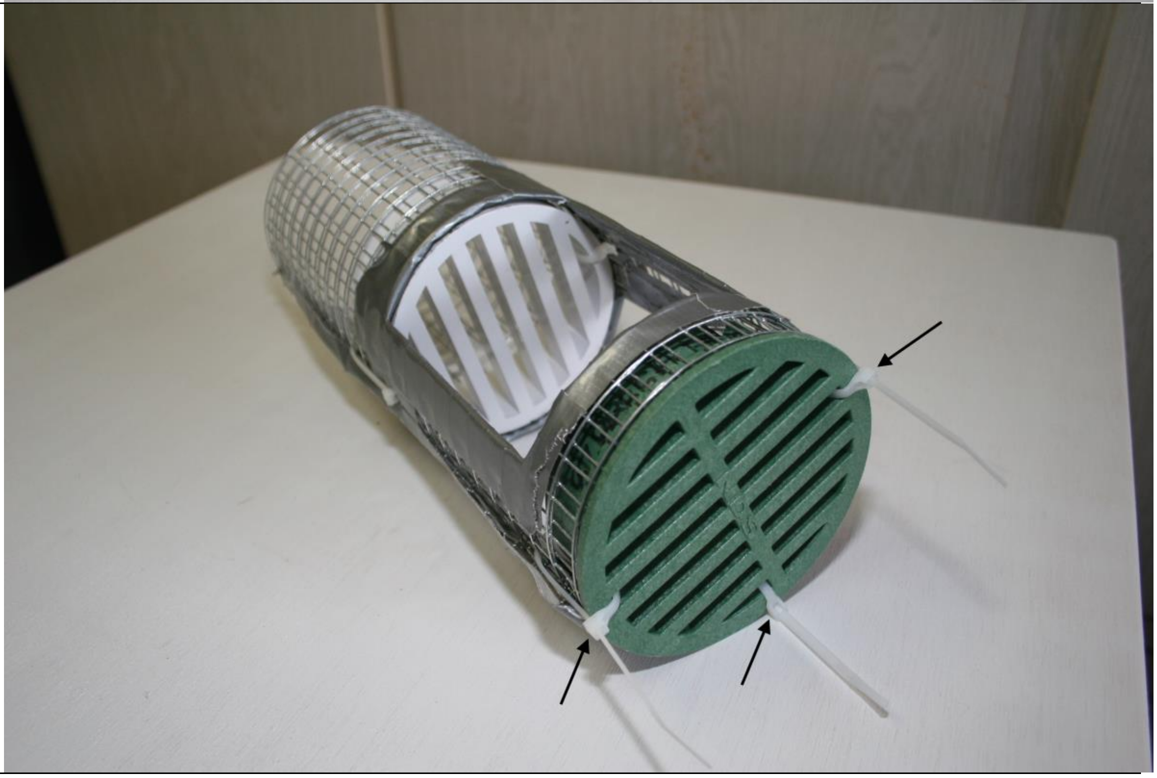

Affix to plywood with utility hook hangers and screws or similar (2d)		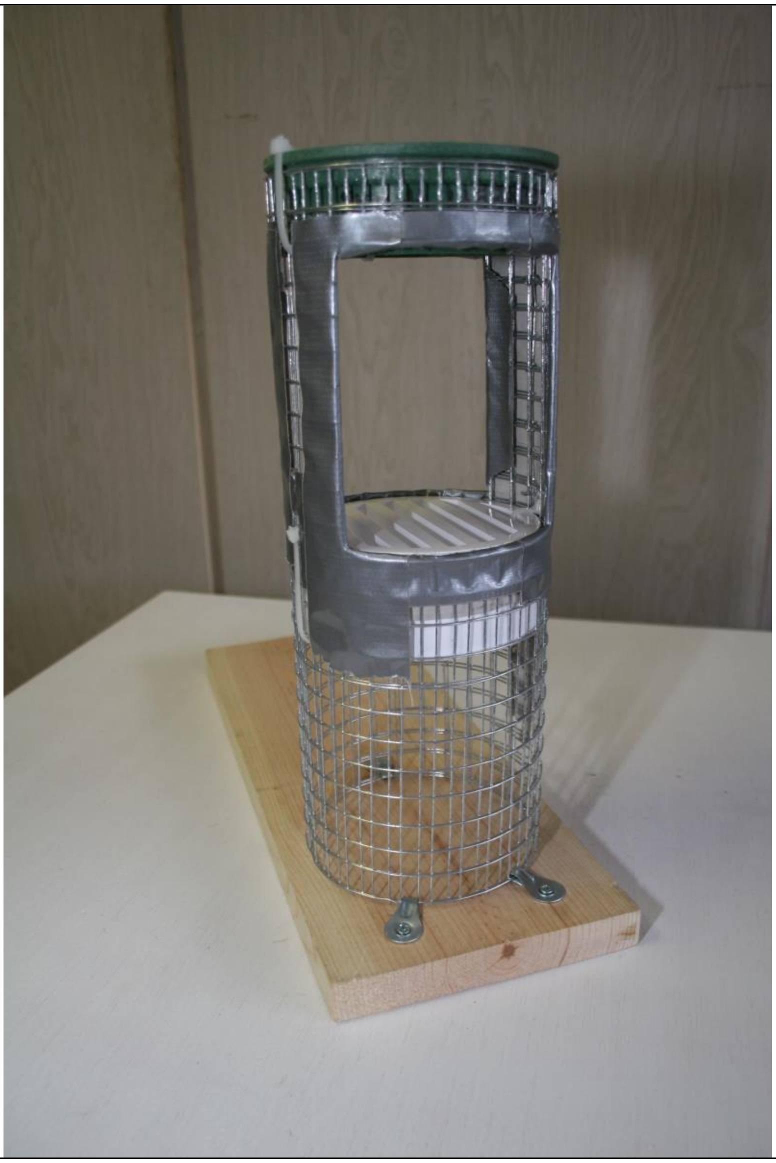

		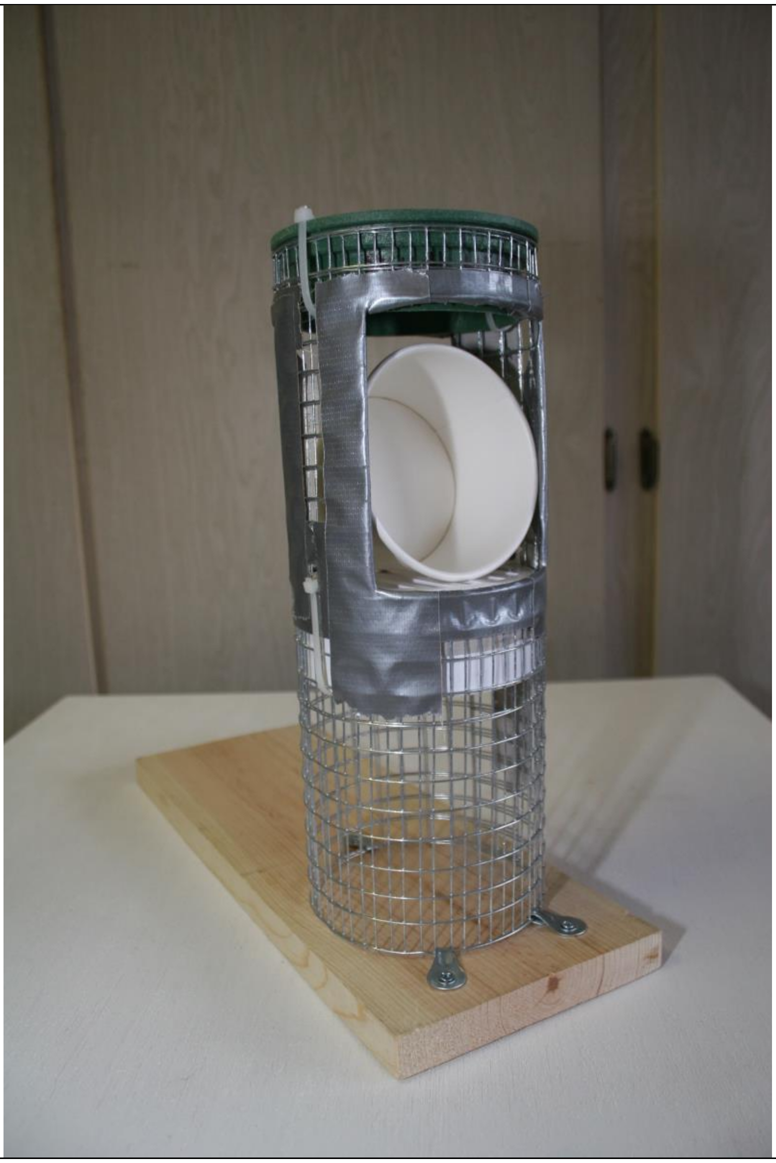

Secure cylinder to headskin with zip ties (2d)	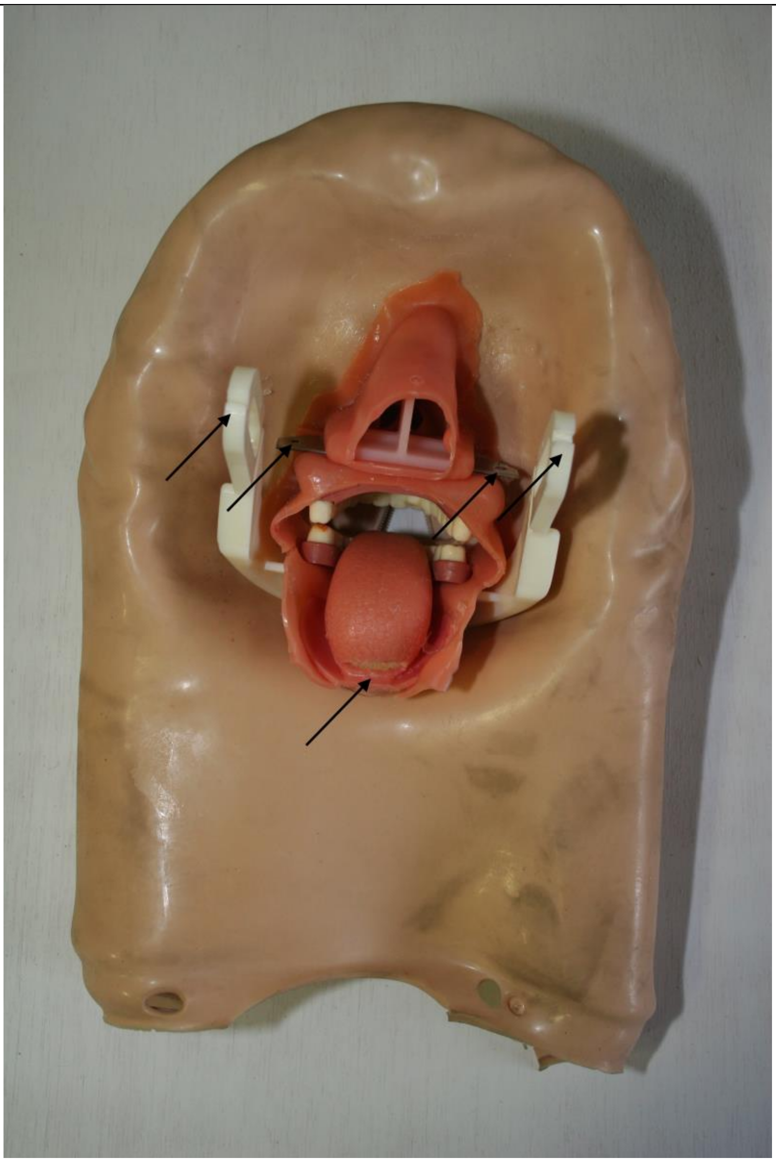 Internal view with arrows marking zip tie locations

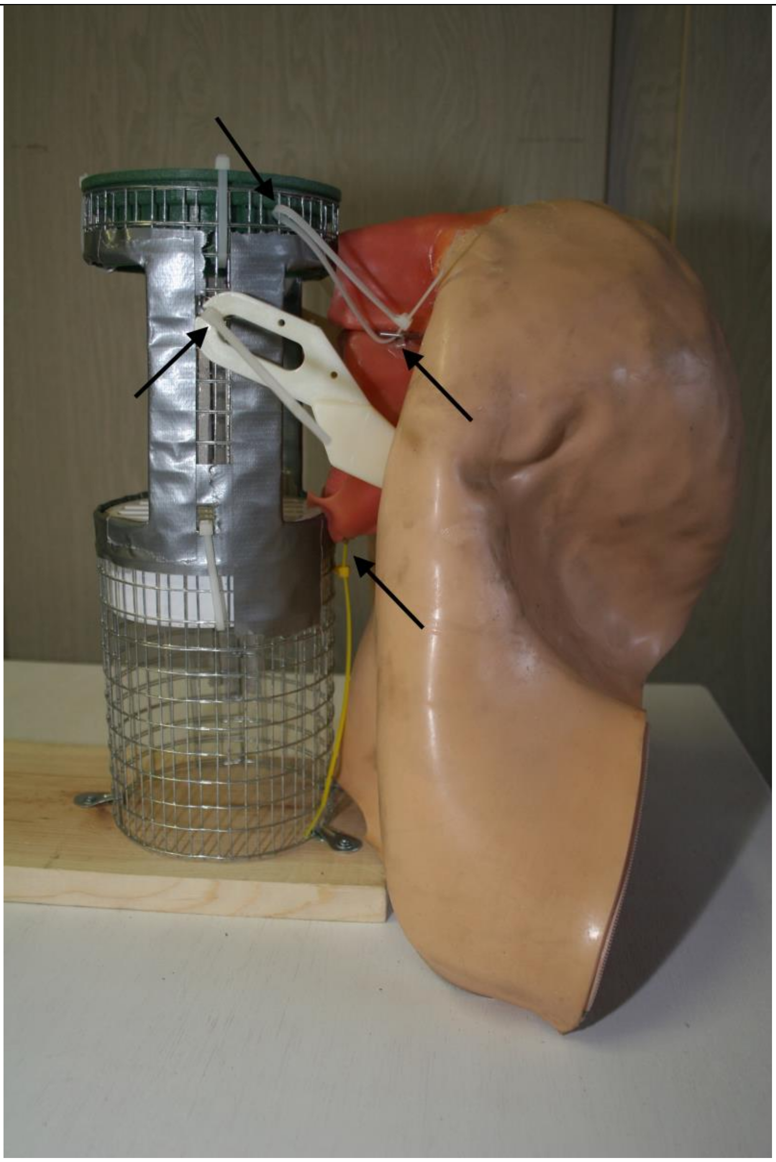

Lateral View, Right Side

	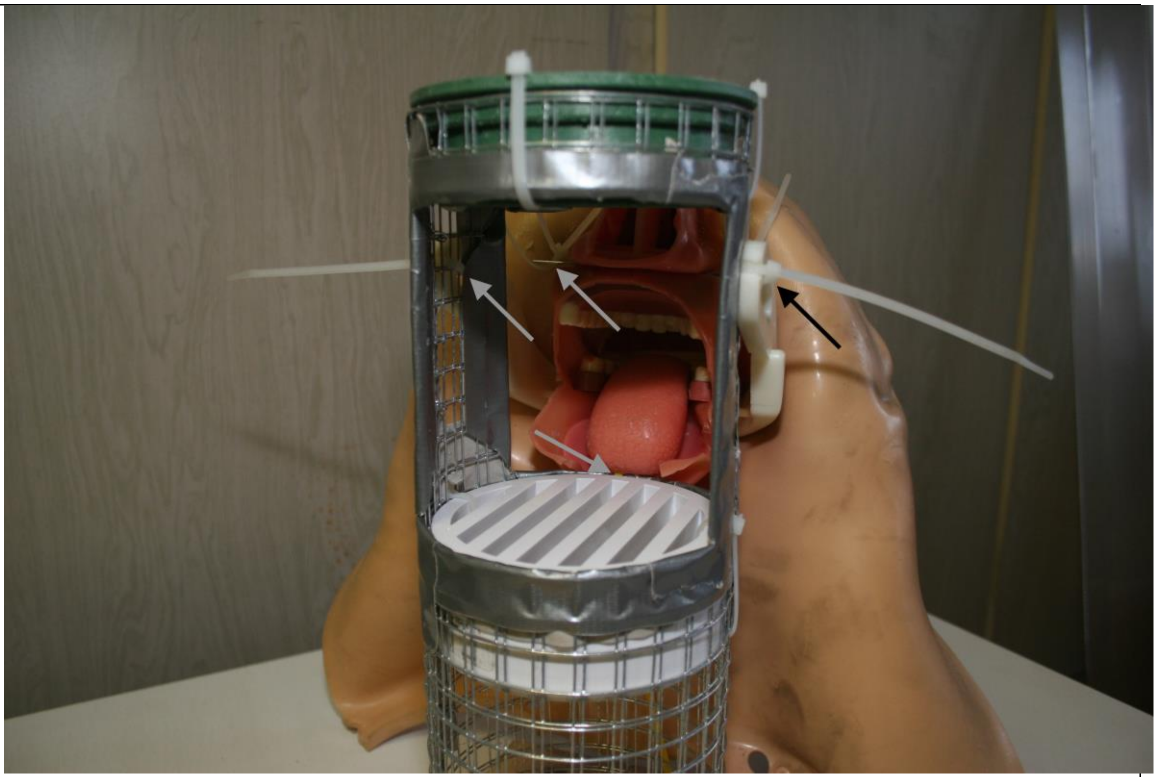<br>Posterior View
Fill with foam or towels to provide structure to head (2d)	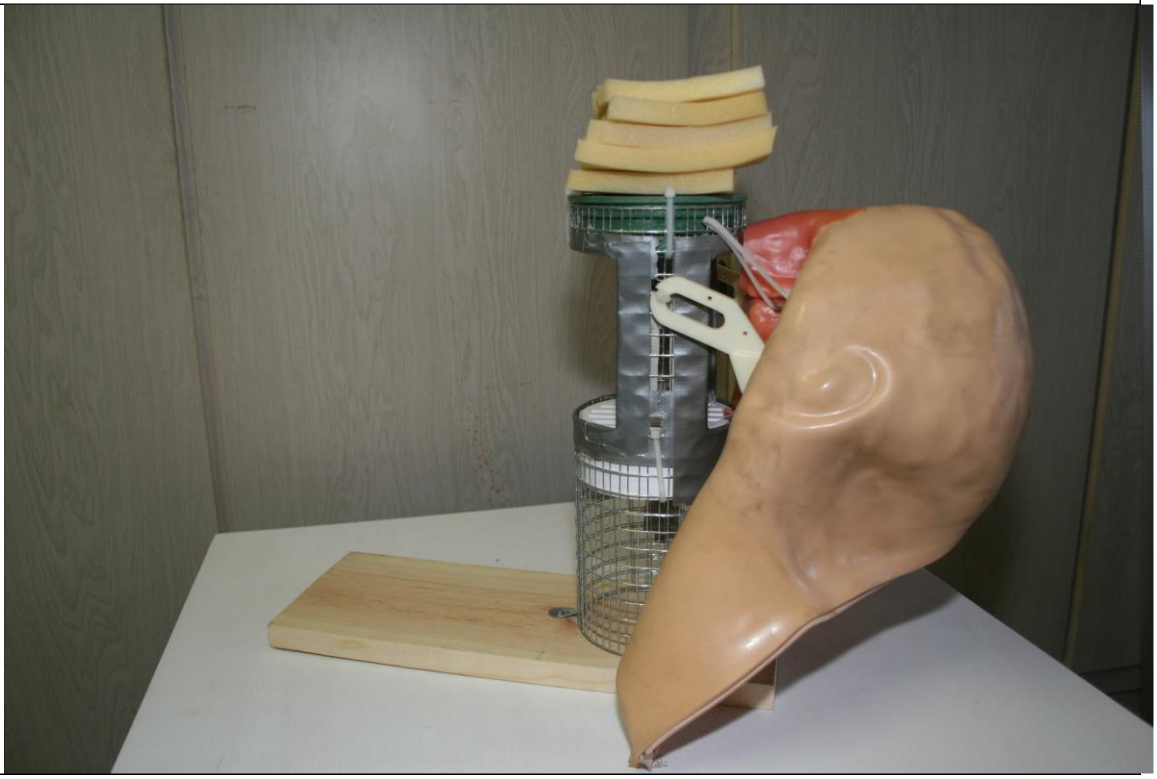

Materials for peritonsillar abscess cups (see Table 1)	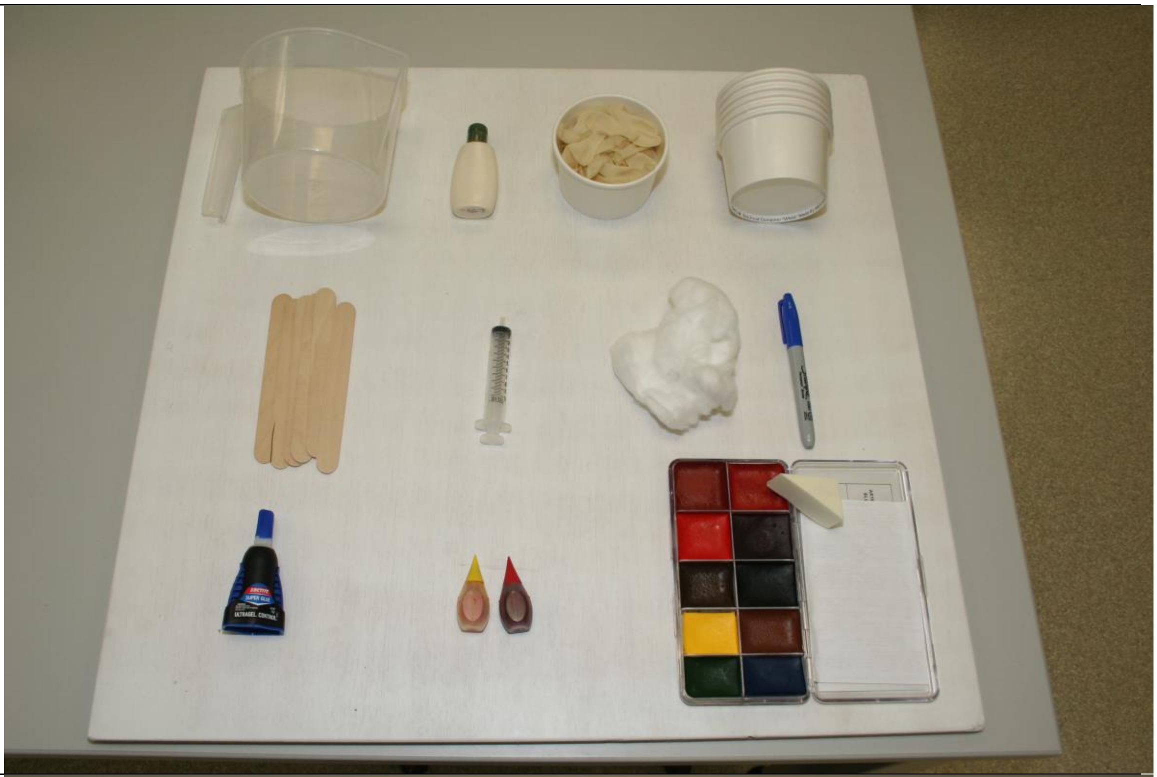
Combine water and barrier lotion to desired viscosity. Ensure material can be aspirated through an 18g spinal needle. (3a)	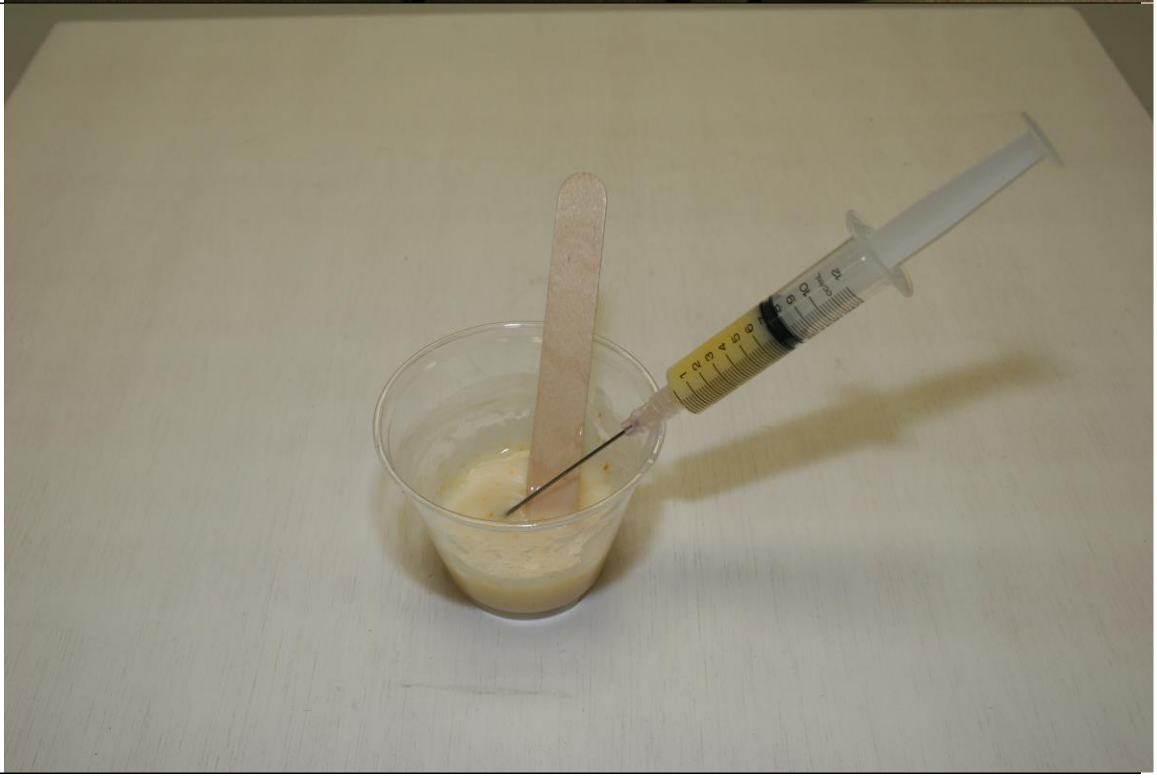

Inject approximately 7ml into small water balloon and tie closed (3a)	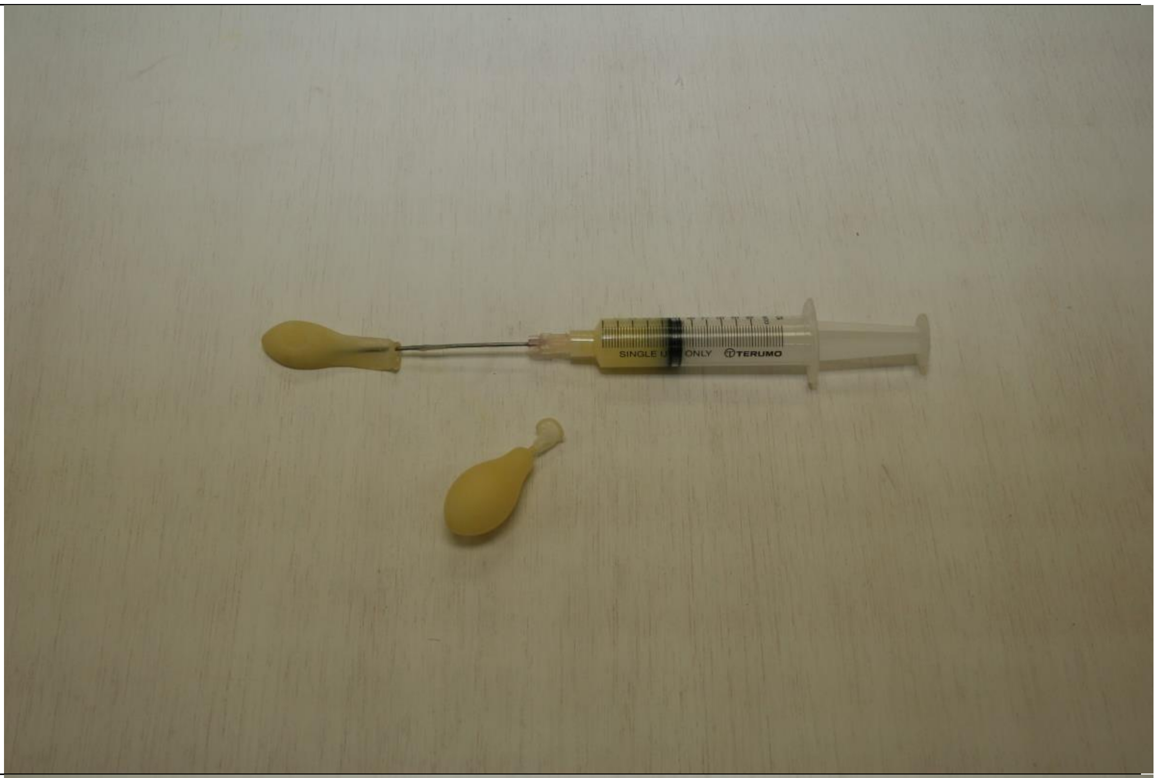
Glue balloon to bottom of cup with cyanoacrylate glue and mark location on cup underside (3b)	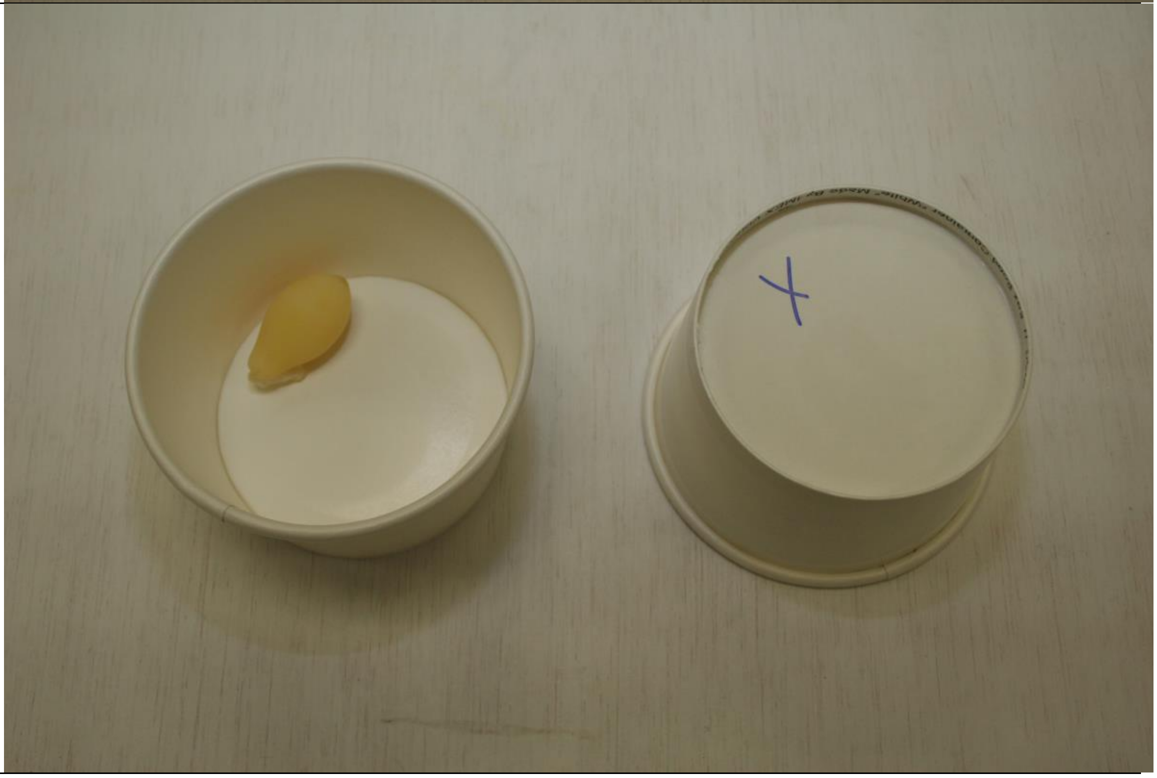

Tape half of a craft stick to inside of cup inferior to abscess to ensure proper orientation in head and cover with cotton (3c)	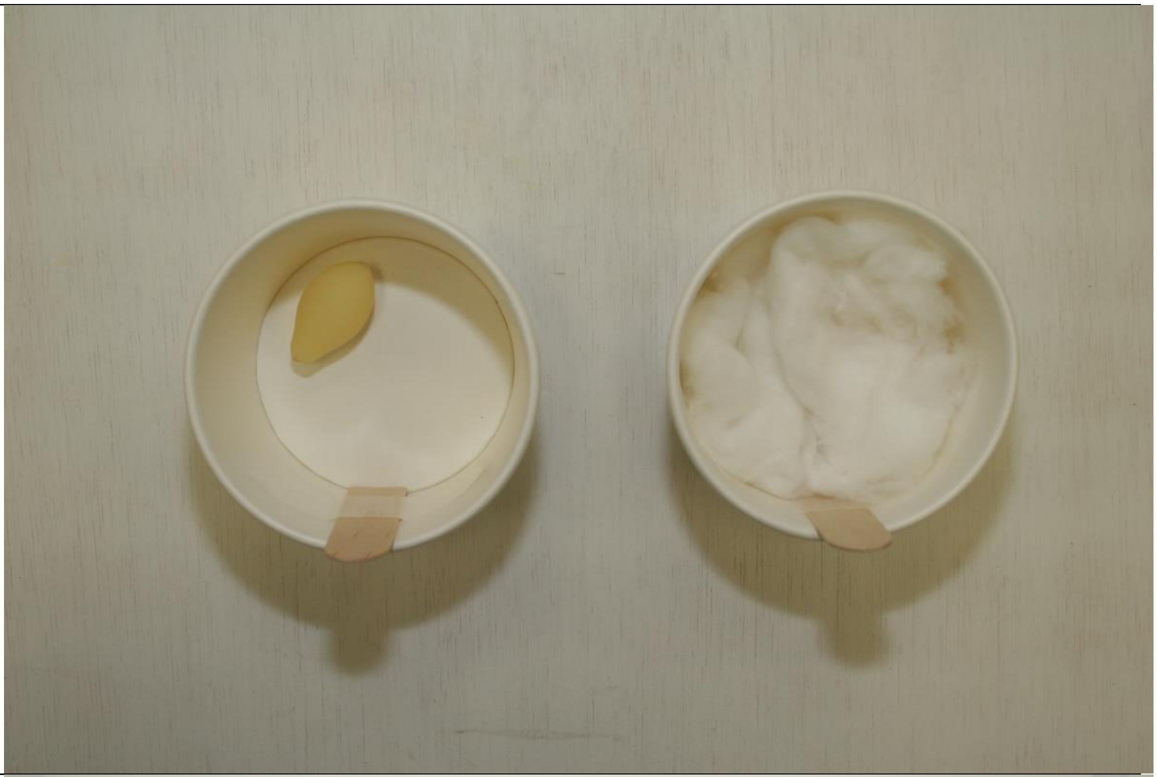
Prepare ballistic gelatin. Combine 100g of gelatin powder per 800ml of water in a glass beaker and stir. Place beaker into heated water bath of at least 75C and allow to sit for a minimum of ten minutes while stirring occasionally.<br><br>Add approximately 1 drop each of red and yellow food coloring per 800ml to achieve desired flesh color. (3d)	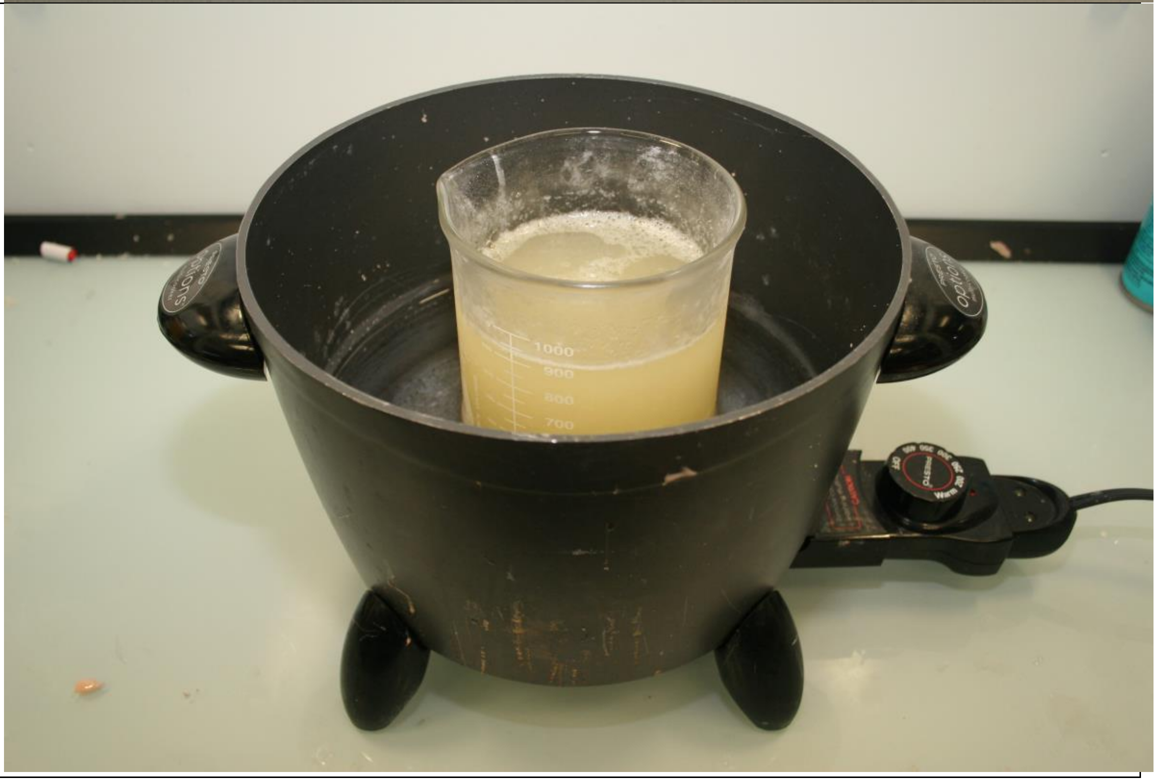

	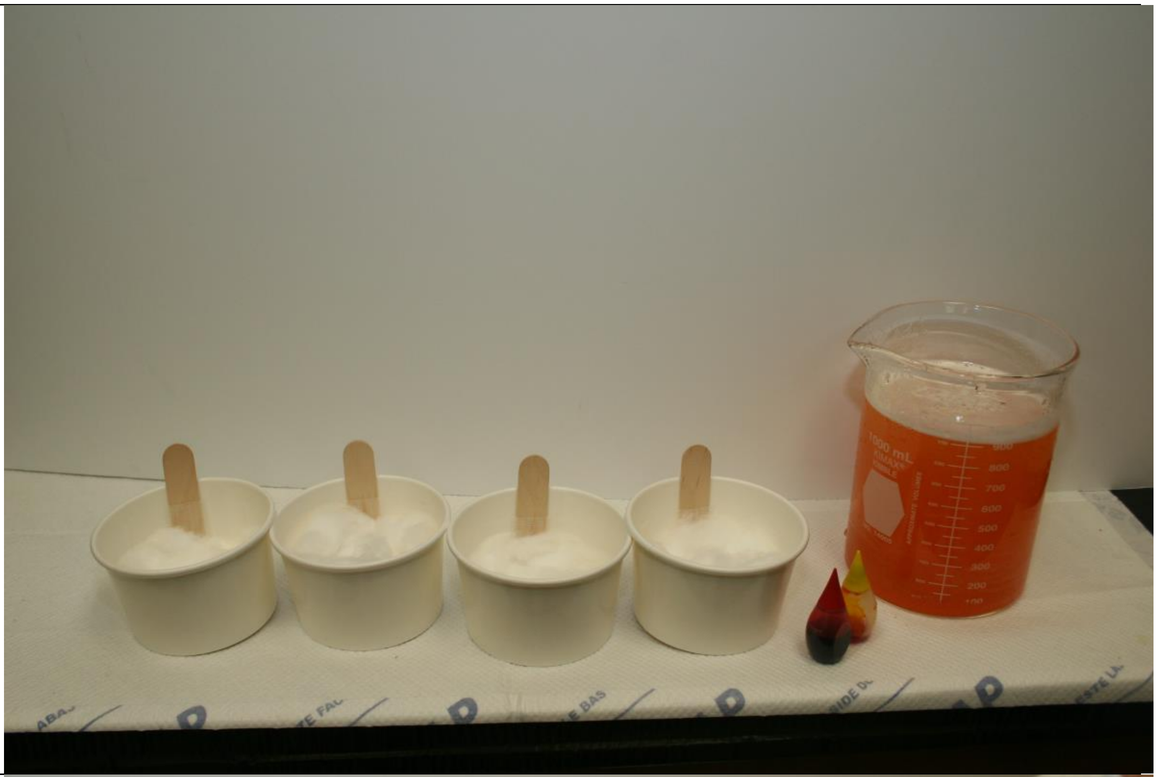
Using heat protective gloves or oven mitts pour gelatin into cup to cover both the balloon and cotton.<br><br>Refrigerate for a minimum of two hours to fully set. (3d)	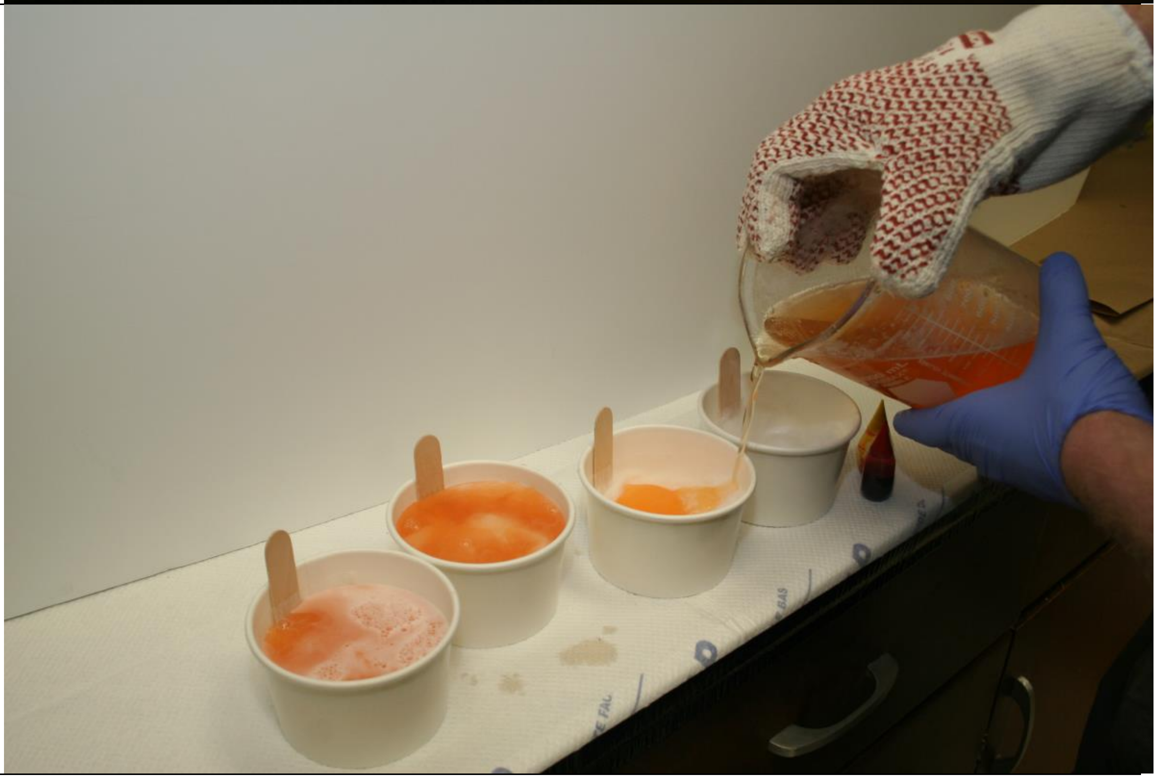

Paint asymmetric soft palate and deviated uvula, corresponding to location of balloon (3e)		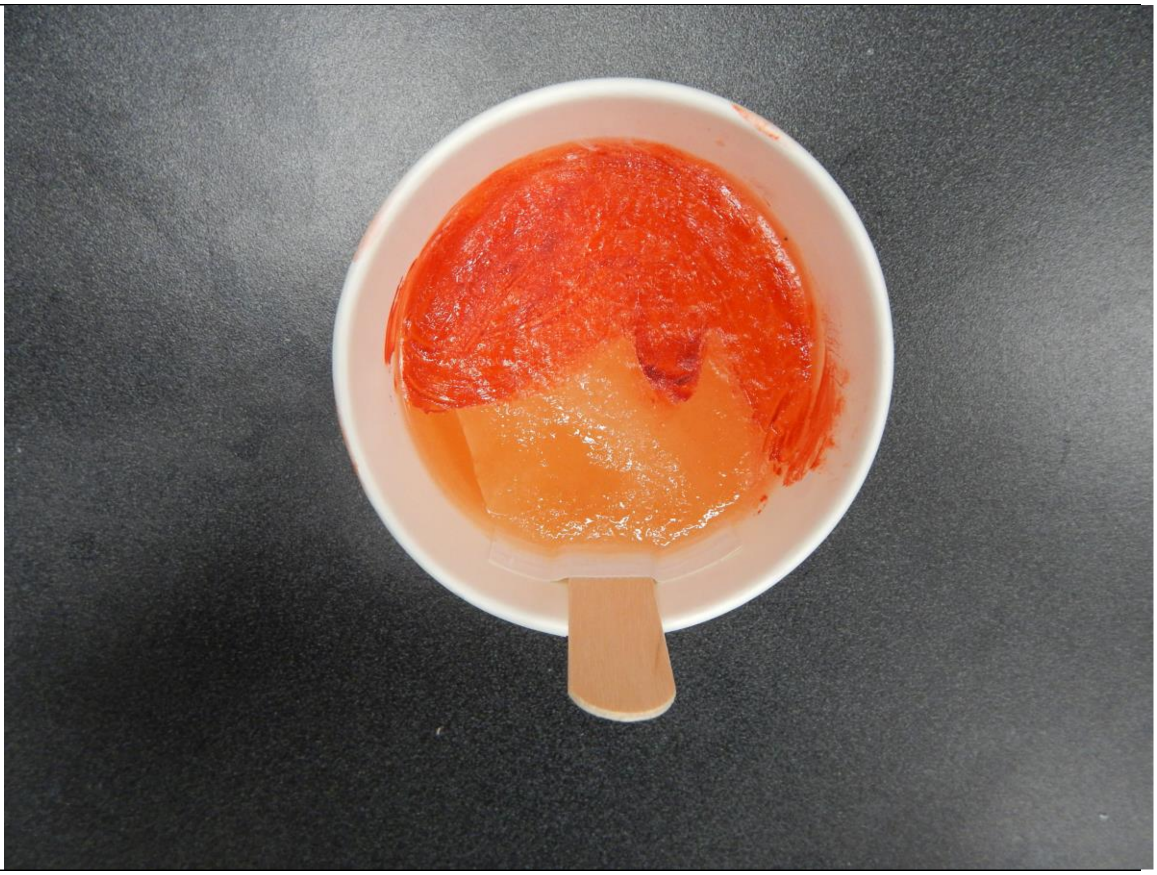

Complete task trainer head with PTA cup in place (4)	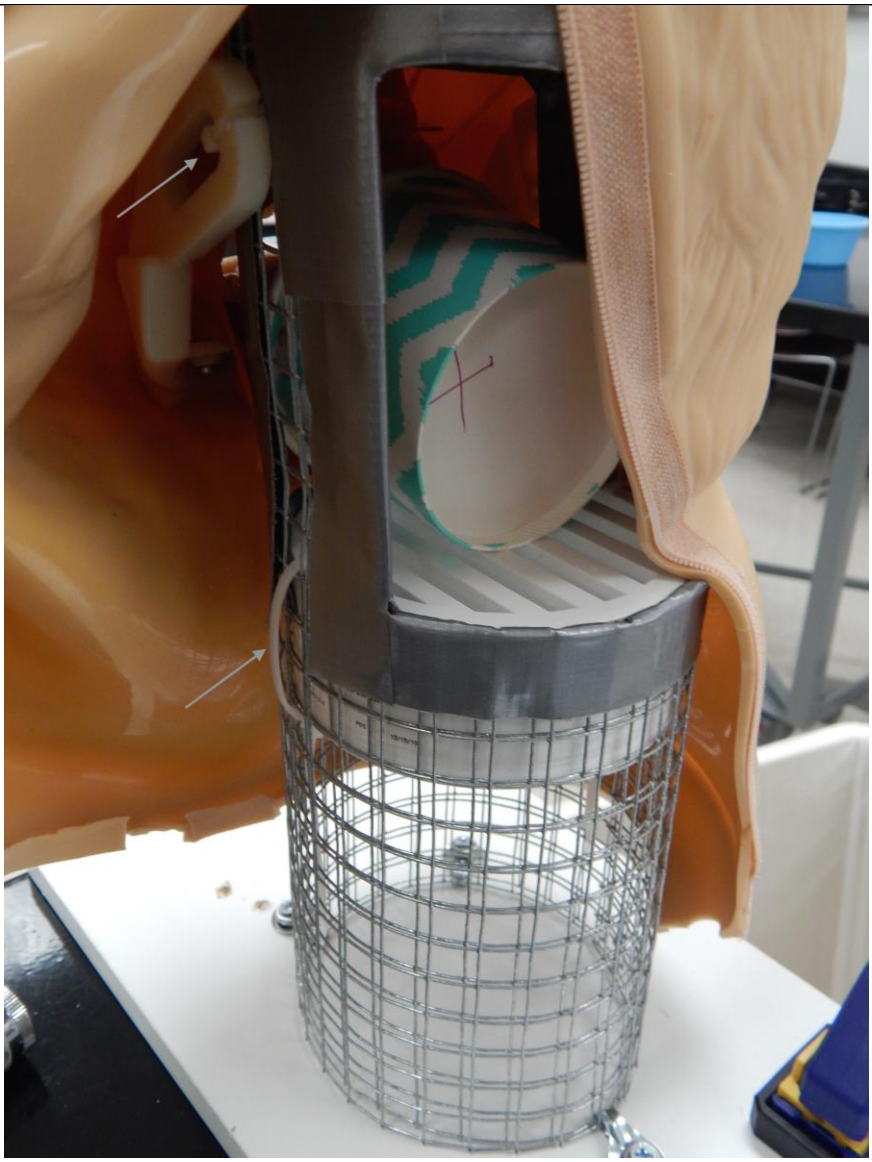<br>Oblique Posterior View

	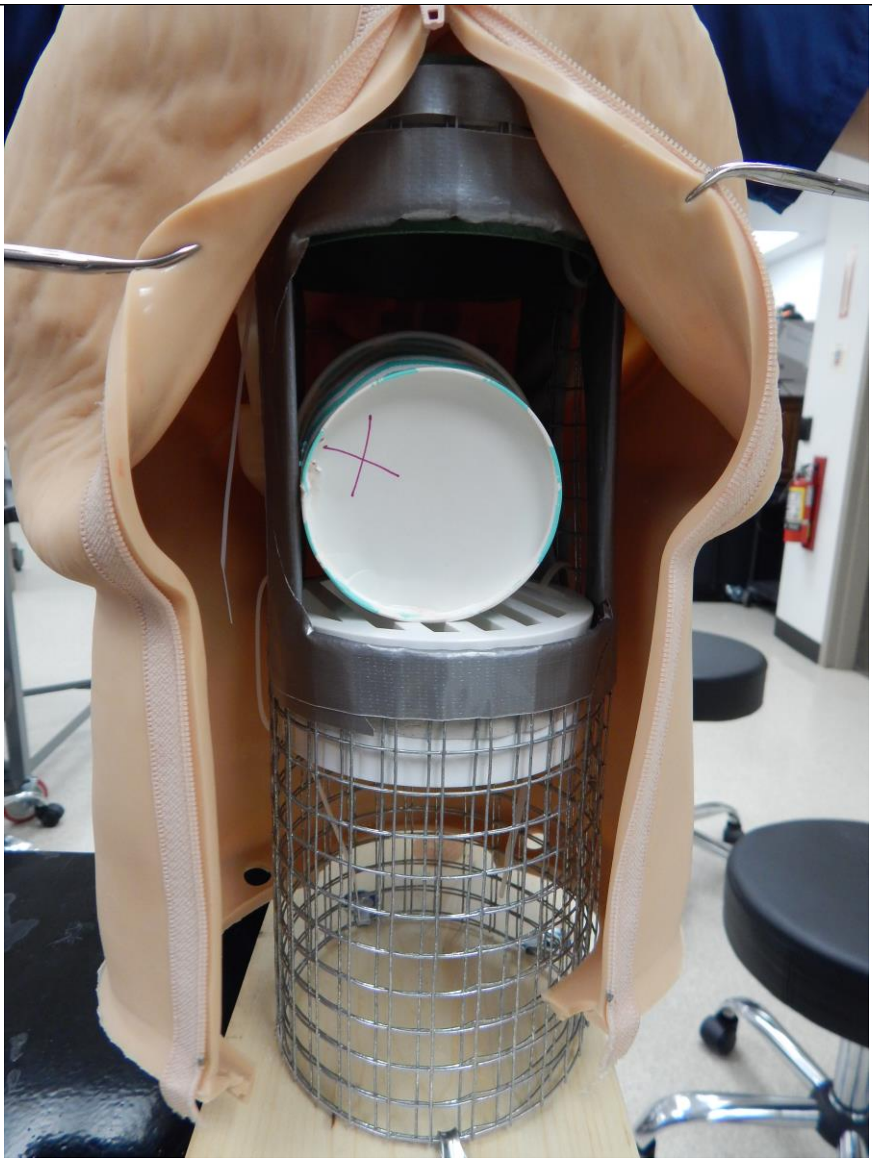 Posterior View	
